# Induced sensorimotor brain plasticity controls pain in phantom limb patients

**DOI:** 10.1038/ncomms13209

**Published:** 2016-10-27

**Authors:** Takufumi Yanagisawa, Ryohei Fukuma, Ben Seymour, Koichi Hosomi, Haruhiko Kishima, Takeshi Shimizu, Hiroshi Yokoi, Masayuki Hirata, Toshiki Yoshimine, Yukiyasu Kamitani, Youichi Saitoh

**Affiliations:** 1 Department of Neurosurgery, Osaka University Graduate School of Medicine, 2-2 Yamadaoka, Suita, Osaka 565-0871, Japan; 2 Division of Functional Diagnostic Science, Osaka University Graduate School of Medicine, 2-2 Yamadaoka, Suita, Osaka 565-0871, Japan; 3 Department of Neuroinformatics, ATR Computational Neuroscience Laboratories, 2-2-2 Hikaridai, Seika-cho, Kyoto 619-0288, Japan; 4 Department of Neuroinformatics, CiNet Computational Neuroscience Laboratories, 2-2 Yamadaoka, Suita, Osaka 565-0871, Japan; 5 JST PRESTO, 2-2 Yamadaoka, Suita, Osaka 565-0871, Japan; 6 Division of Clinical Neuroengineering, Osaka University, Global Center for Medical Engineering and Informactics, 2-2 Yamadaoka, Suita, Osaka 565-0871, Japan; 7 Graduate School of Information Science, Nara Institute of Science and Technology, 8916-5 Takayamacho, Ikoma, Nara 630-0192, Japan; 8 Department of Engineering, University of Cambridge, Computational and Biological Learning Laboratory, Trumpington Street, Cambridge CB2 1PZ, UK; 9 National Institute for Information and Communications Technology, Center for Information and Neural Networks, 1-3 Suita, Osaka 565-0871, Japan; 10 Department of Neuromodulation and Neurosurgery, Osaka University Graduate School of Medicine, 2-2 Yamadaoka, Suita, Osaka 565-0871, Japan; 11 Department of Mechanical Engineering and Intelligent Systems, The University of Electro-Communications, 1-5-1 Chofugaoka, Chofu, Tokyo 182-8585, Japan; 12 Graduate School of Informatics, Kyoto University, Yoshidahonmachi, Sakyoku, Kyoto 606-8501, Japan

## Abstract

The cause of pain in a phantom limb after partial or complete deafferentation is an important problem. A popular but increasingly controversial theory is that it results from maladaptive reorganization of the sensorimotor cortex, suggesting that experimental induction of further reorganization should affect the pain, especially if it results in functional restoration. Here we use a brain–machine interface (BMI) based on real-time magnetoencephalography signals to reconstruct affected hand movements with a robotic hand. BMI training induces significant plasticity in the sensorimotor cortex, manifested as improved discriminability of movement information and enhanced prosthetic control. Contrary to our expectation that functional restoration would reduce pain, the BMI training with the phantom hand intensifies the pain. In contrast, BMI training designed to dissociate the prosthetic and phantom hands actually reduces pain. These results reveal a functional relevance between sensorimotor cortical plasticity and pain, and may provide a novel treatment with BMI neurofeedback.

## Abstract (web summary)

Pain in a phantom limb after limb deafferentation may be due to maladaptive sensorimotor representation. Here the authors find that sensorimotor plasticity induced by BMI training with the phantom hand, contrary to expectation, increased pain while dissociating prosthetic movements from the phantom arm relieved the pain.

## Graphical abstract

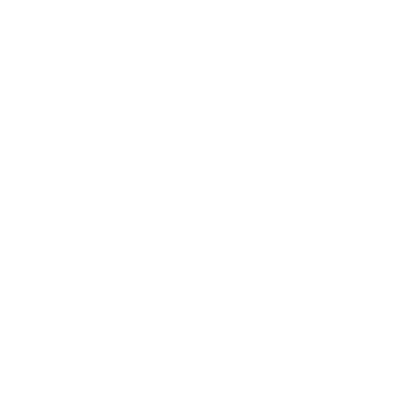

Phantom limb pain describes the intractable chronic pain^1^ that frequently occurs in a partially or completely deafferented body part after severe peripheral nerve injury^2^ or amputation^3^. A prominent theory is that the underlying cause of the pain is maladaptive plasticity of the sensorimotor cortex^3^,^4^,^5^. The key evidence supporting this is the correlation between pain and topographic reorganization of sensorimotor cortical maps, as revealed by several methods^6^,^7^,^8^. Furthermore, rehabilitative therapies inspired by the theory, such as discrimination training, myoelectric prosthesis use, nerve replantation and mirror therapy, reduce pain and show a corresponding correlation with topographic reorganization^5^,^9^,^10^,^11^,^12^. This underlies a widely held guiding principle that restoration of function reduces pain^3^.

However, recent evidence has caused researchers to question the maladaptive sensorimotor reorganization model, especially in terms of the motor representation of the phantom limb. For example, it has been shown that when using ‘unfolded' brain maps to study anatomical reorganization of the phantom hand, shifts in the peaks of lip representations are local, do not invade the hand area and are not correlated with pain^13^. Furthermore, functional brain activity in the area of missing hands of amputees does not increase during lip movements^14^. In the phantom symptoms associated with nerve loss in carpal tunnel syndrome, sensorimotor reorganization is correlated with paraesthesia, but not pain^15^. Even the efficacy of behavioural therapies designed to target reorganization has been questioned^11^. Critically, however, both sides of this argument lack any direct experimental manipulation of sensorimotor plasticity that, in principle, could be used to support or refute a causative link between sensorimotor cortical plasticity and pain.

Recently, it has been shown that brain–machine interfaces (BMIs) can not only reconstruct motor function in severely paralysed patients^16^ but also induce plastic changes in cortical activity^17^,^18^,^19^. A BMI works by first decoding neural activity of the mental action to move the affected hand, for example, and then by converting the decoded phantom hand movement into that of a robotic neuroprosthesis. BMIs based on magnetoencephalography (MEG) sensorimotor cortex signals have been shown to be sufficient to precisely decode hand movements in real time^20^,^21^, even in severely paralysed patients simply intending to move the affected hands^22^,^23^. Moreover, training to use BMIs induces plastic changes in cortical activity^24^,^25^ and, potentially, associated clinical symptoms^26^.

Here we apply the BMI training of a neuroprosthetic (robotic) hand using real-time MEG signals in phantom limb patients, whose symptoms are primarily caused by nerve avulsion, and evaluate the association between changes in their symptomatic pain and in cortical currents during phantom hand movements. We show that the patients partially restore the function of the affected hand by using the prosthetic hand in its place. According to the principle of restoration of function, it is initially hypothesized that successful BMI training using decoded phantom hand movements should reduce pain with concurrent plastic changes in cortical activity. However, our results show that, although BMI training leads to a significant increase of movement information in the sensorimotor cortex, the training significantly increases pain. On the other hand, BMI training to associate the prosthetic hand with the intact hand representation reduces pain with decreased information about phantom hand movements. These results suggest a causative relationship between sensorimotor cortical plasticity and pain.

## Results

### Experimental design of BMI training

**Figure 1:**
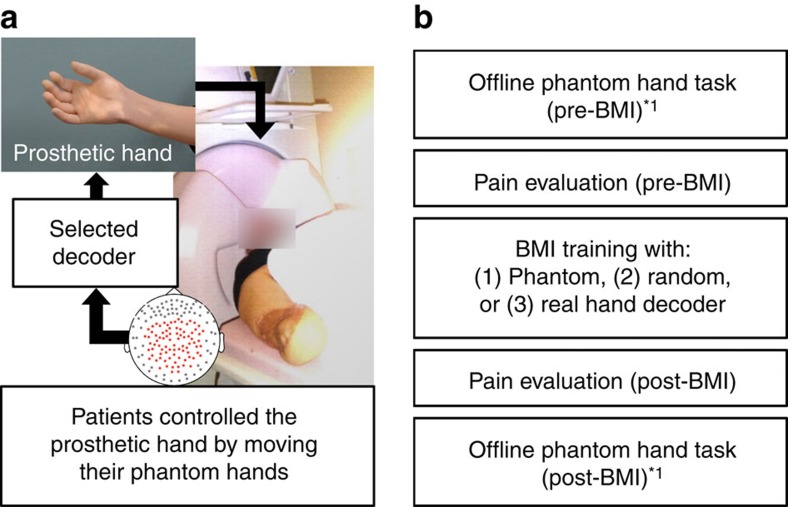
BMI training and experimental design. (**a**) BMI training. Patients were instructed to control the prosthetic hand by moving their phantom hands in each experiment. (**b**) A diagram of the tasks in each experiment. First, the patients performed the offline phantom hand task to move their phantom hand according to the instructions. Then, after evaluation of their pain, BMI training was performed for 10 min. Here three types of decoders were used to control the prosthetic hand, each for three experiments. After evaluation of their pain, the same offline phantom hand task was performed. *^1^For the experiment with the real hand decoder, the patients also performed the offline task with their intact hand after the task with their phantom hand.

BMI training was applied to 10 patients with phantom limb due to brachial plexus root avulsion (*n*=9) and amputation of the right forearm (*n*=1; Table 1 and Supplementary Table 1)^20^. All patients had phantom limb symptoms, and the associated pain is consequently thought to share a common mechanism with phantom limb pain due to amputation^3^. All patients participated in BMI training to control the robotic hand (Fig. 1a). Cortical plasticity was evaluated by comparing the same offline task to move the phantom hand before and after each training session (Fig. 1b). At the same time, pain was evaluated with a visual analogue scale (VAS) and the Japanese version of the short-form McGill Pain Questionnaire 2 (SF-MPQ2)^27^.

### Decoding of the phantom hand movement using MEG signals

**Figure 2:**
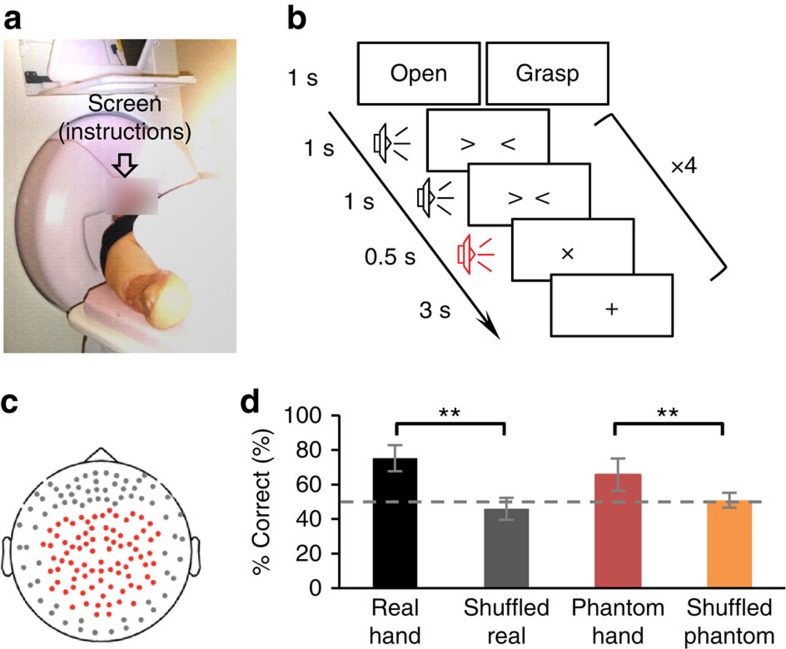
Offline evaluation of movements of real hands and phantom hands. (**a**) Schematic figure representing the offline task. The patients intended to move their phantom hand according to the instruction presented on the screen in front of them. (**b**) Experimental paradigm. An epoch began with a 3-s visual presentation of a black cross. A Japanese word was shown for 1 s to instruct the subjects which movement to perform. After two 1-s timing cues, the execution cue of the cross sign was presented for 0.5 s with a sound. The patients performed the instructed movements once when the execution cue was presented. These cues with sounds were repeated four times for each instruction. Each of the movement types was assigned in random order 10 times each. (**c**) We recorded the MEG signals of the 84 selected sensors, which are shown as red points on the picture of the sensor location. (**d**) The average classification accuracy of movement types using the *z-*scored MEG sensor signals (mean and 95% confidence interval). Real hand (black), phantom hand (red) and the randomly relabelled data of the real hand (grey) and phantom hand (orange; *n*=10). **P<0.01, Bonferroni-corrected, two-tailed Student's *t-*test.

First, we performed an ‘offline' task (pre-BMI) in which patients were instructed to try to move their phantom hands to be in the posture of grasping or opening according to given instructions (Fig. 2a,b)^16^. The patients could not see their actual arms during the experiment. The MEG signals from 84 selected sensors were recorded during the task (Fig. 2c) and then time-averaged using windows of 500 ms slid by 100 ms, in the range from −500 to 1,000 ms with respect to the timings of the execution cues. Then, the averaged signals were converted into *z*-scores using the mean and s.d. estimated from the initial 50 s of the offline task.

A nested 10-fold cross-validation^28^ was performed with a support vector machine (SVM) to evaluate the accuracy of classifying the performed movement types using the *z*-scores of the selected MEG sensors^20^. The accuracy was evaluated for each 500-ms window, and the maximum value of the accuracies was estimated as the classification accuracy. The classification accuracy of the phantom movement during the pre-BMI task was 65.6±9.4% (mean±95% confidence interval, *n*=10), which was significantly greater than the accuracies for classifying the same signals with randomly relabelled movement types (50.9±4.3%, *P*=0.0012, Bonferroni-corrected, *t*(18)=2.79, two-tailed Student's *t-*test, *n*=10; Fig. 2d).

For comparison, the patients performed actual movements of their intact hands according to the same instructions during another experiment. The classification accuracy was evaluated by the same method. For the intact hand movements before the BMI training, the classification accuracy was 75.3±7.5% (mean±95% confidence interval, *n*=10), which exceeded the accuracies of randomized movement types (46.0±6.3%, *P*=0.000016, Bonferroni-corrected, *t*(18)=5.83, two-tailed Student's *t-*test, *n*=10; Fig. 2d). Notably, the classification accuracies were comparable between the phantom movements and the intact hand movements (*n*=10, *P*=0.13, Bonferroni-corrected, *t*(18)=1.57, two-tailed Student's *t-*test), although the electromyographic signals of the affected hands were not comparable to those of the intact hands (Supplementary Fig. 1).

### Estimated cortical currents

**Figure 3:**
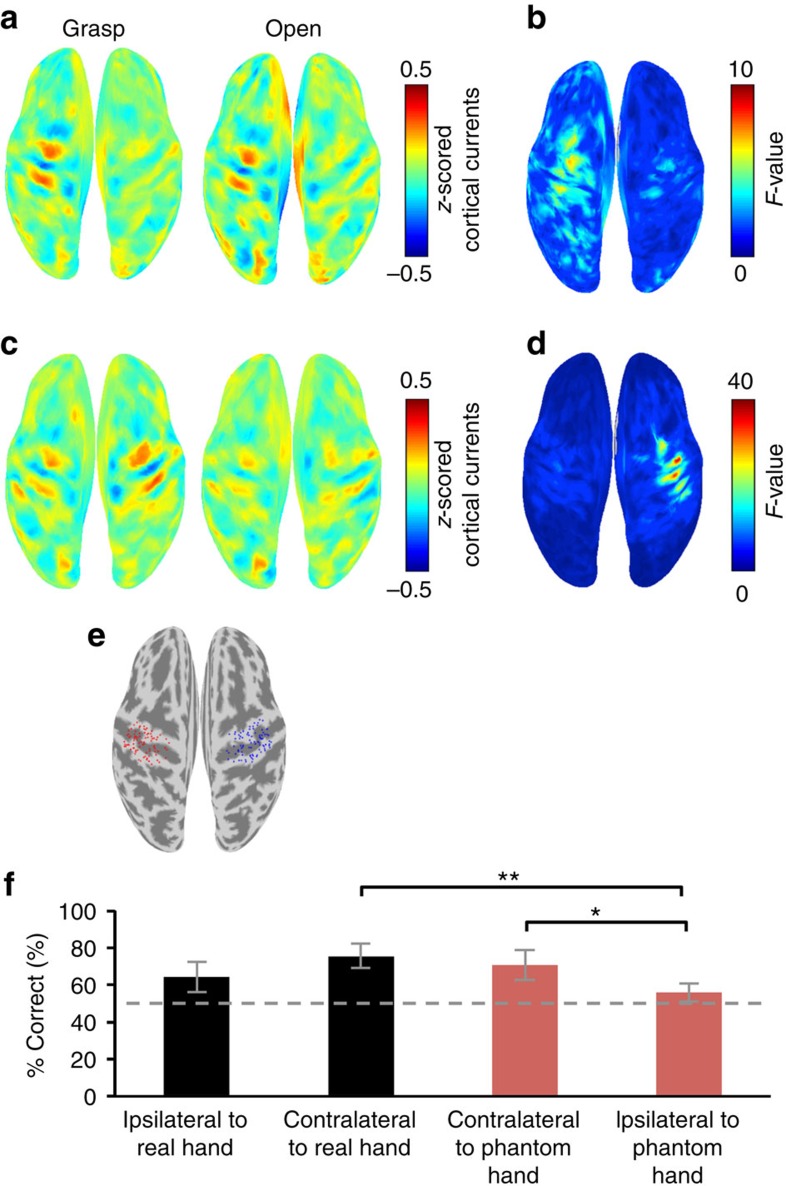
Estimated cortical currents and the decoding of movements. (**a**) The mean *z-*scored cortical currents during grasping and opening of the phantom hand were colour-coded on a normalized brain surface at the time of movement onset (the side of the phantom hand is shown on the right, *n*=10). (**b**) The mean *F-*values of ANOVA of the *z-*scored cortical currents between the two movements were colour-coded on the normalized brain surface (the side of the phantom hand is shown on the right, *n*=10). (**c**) The mean *z-*scored cortical currents during grasping and opening of the real hand (*n*=10). (**d**) The mean *F-*values of ANOVA of the *z-*scored cortical currents between the two movements of the real hand (*n*=10). (**e**) The 126 vertices were selected in the sensorimotor cortex contralateral (red) and ipsilateral (blue) to the phantom hand. (**f**) The classification accuracy of movement types using the *z-*scored cortical currents on the sensorimotor cortex is shown with the 95% confidence interval for each hemisphere of each hand (*n*=10; black, real hand; red, phantom hand). The asterisks denote significant differences (*P<0.05, **P<0.01, Bonferroni-corrected, two-tailed Student's *t-*test). The dashed, grey line indicates the accuracy by chance (50%).

We evaluated the cortical representation during the offline task using cortical current source estimation and decoding accuracy. First, the cortical currents were estimated from the obtained MEG signals using variational Bayesian multimodal encephalography (VBMEG)^29^. Next, the estimated cortical currents were averaged using a 500-ms time window from the execution cue and *z*-scored by the estimated cortical currents of the initial 50-s period of the offline session. The *z*-scored cortical currents were averaged for the 10 patients and colour-coded on the normalized brain surface. The right hand was designated as the affected hand; for those two patients with affected left hands, the cortical activation was switched. Figure 3a shows the cortical currents activated on the sensorimotor cortex contralateral to the phantom hand when the patients attempted to move their phantom hands.

The *z*-scored cortical currents were compared between two types of movements with a one-way analysis of variance (ANOVA) for each vertex. The *F*-value of the ANOVA was averaged for the 10 patients and colour-coded on the normalized brain surface. Figure 3b shows that cortical currents on the contralateral sensorimotor cortex varied between movement types with high *F*-values (see also Supplementary Fig. 2).

Similarly, for the intact hand movements, the cortical currents were activated on the sensorimotor cortex contralateral to the moved hand, depending on the movement types, with high *F*-values (Fig. 3c,d). The cortical currents on the contralateral sensorimotor cortex varied according to the movement types for both the real and the phantom hand.

Next, the decoding method was applied for the *z*-scored cortical currents to quantify how accurately the cortical currents represent the two types of phantom movements. A total of 126 vertices were selected on the sensorimotor cortex of each hemisphere (Fig. 3e). The *z*-scored cortical currents were estimated at the selected vertices from −500 to 1,000 ms for each 100 ms. Using the same method for the *z-*scored MEG sensor signals, the *z*-scored cortical currents were evaluated to determine the accuracy for classifying the movement types.

In all patients, the classification accuracies varied significantly between each side of the sensorimotor cortex for the movements of the real and phantom hands (*n*=10 each, *P*=0.0032, *F*(3, 36)=5.52, one-way ANOVA; Fig. 3f). The accuracy for classifying the phantom movement was significantly higher using the currents on the sensorimotor cortex contralateral to the phantom hand compared with the currents on the ipsilateral cortex (*n*=10, *P*=0.007, Bonferroni-corrected, *t*(18)=3.06, two-tailed Student's *t-*test; Fig. 3f). Thus, the classifier clearly distinguished the information on the ipsilateral and contralateral hemispheres. Moreover, the accuracy of the real hand movement with the contralateral sensorimotor cortex was significantly higher than the accuracy of the phantom movement with the same hemisphere, ipsilateral to the phantom hand (*n*=10, *P*=0.00017, Bonferroni-corrected, *t*(18)=4.72, two-tailed Student's *t-*test; Fig. 3f). This indicates the specificity of motor information represented in the sensorimotor cortex.

The classification accuracy using the cortical currents contralateral to the moved hand was not significantly different between the phantom hand and real hand (*n*=10, *P*=0.37, Bonferroni-corrected, *t*(18)=0.93, two-tailed Student's *t-*test). Moreover, the classification accuracies of the phantom movements before BMI training were not significantly different in any experiment (*n*=10 each, *P*=0.75, *F*(2, 27)=0.29, one-way ANOVA; Supplementary Fig. 3). Thus, in summary, the representation of the hand's movement was preserved in the contralateral sensorimotor cortex, even for phantom movements.

### BMI training with a neuroprosthetic hand

The BMI training to control the robotic hand was performed as a randomized crossover trial consisting of two training sessions on two different days (Fig. 1b). Each training session was performed with two different decoders to control the robotic hand: a phantom decoder and a random decoder. Using the *z-*scored MEG sensor signals of the offline tasks to move the phantom hand, we constructed the phantom decoder to infer phantom hand movements at an arbitrary time in order to control the robotic hand in real time^20^. In contrast, the random decoder was constructed from the MEG signals of the same task with randomly relabelled movement types.

For training with either decoder, the patients were instructed to freely control the neuroprosthetic hand for grasping and releasing a ball by trying to move their phantom hands while watching the movement of the prosthetic hand in closed-loop conditions (Supplementary Movie 1 and Fig. 1a). The neuroprosthetic hand was controlled according to the movements inferred by a selected decoder with the *z-*scored MEG sensor signals obtained online. The patients performed the experiments twice with each decoder selected randomly for the crossover portion of the trial without knowing the type of the decoder.

After each experiment we asked the patients about their feelings during controlling the neuroprosthesis. Nine patients reported improvement of their control after training with the phantom decoder (Supplementary Table 2).

### Effects of BMI training on cortical currents and pain

**Figure 4:**
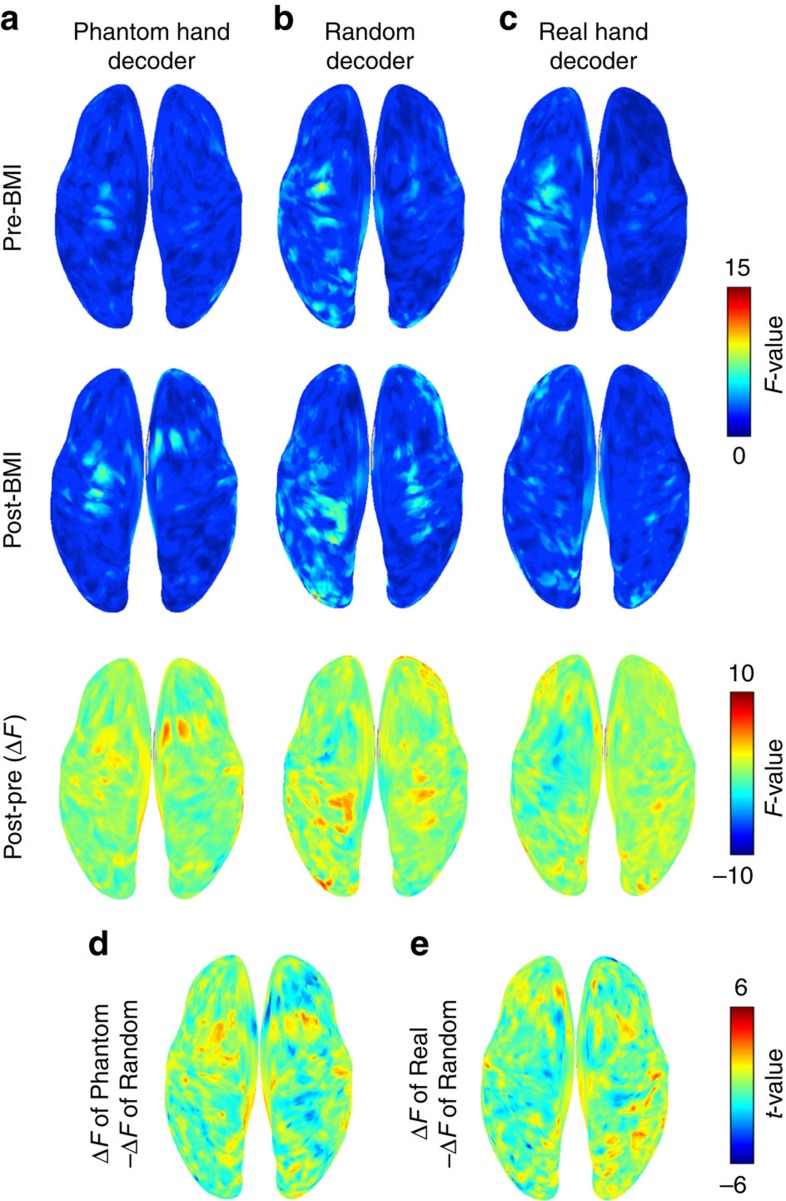
Sensorimotor plasticity induced by BMI training. (**a**–**c**) Among 10 patients, the *F*-value for the two phantom movements was averaged at each vertex and colour-coded on the normalized brain surfaces for the pre-BMI (upper panel), post-BMI (middle panel) and the difference between the pre-BMI and post-BMI (lower panel). Each raw image corresponds to each experiment: (**a**) phantom hand decoder; (**b**) random decoder; and (**c**) real hand decoder. (**d**,**e**) The difference in the *F*-value between pre-BMI and post-BMI compared with that of the random decoder. (**d**) ΔF of the phantom hand decoder−ΔF of the random decoder; (**e**) ΔF of the real hand decoder−ΔF of the random decoder (uncorrected paired Student's *t-*test).

For each offline task, the *F*-values of the *z-*scored cortical currents at the execution cue were averaged across the 10 patients (Fig. 4a–c). After BMI training with the phantom decoder, the *F*-values increased in the contralateral sensorimotor cortex (Fig. 4a). Surprisingly, the pain scores also significantly increased from 38.2±18.5 (mean±95% confidence interval) to 45.8±18.4 in VAS (1/100; *n*=10, *P*=0.0066, uncorrected, *t*(9)=3.51, paired Student's *t-*test), whereas the total scores of the SF-MPQ2 did not significantly increase, changing from 20.4±15.2 to 23.8±17.1 (*n*=10, *P*=0.086, uncorrected, Wilcoxon signed-rank test).

On a different day, the same patients were trained with a random decoder. Using this decoder, the classification accuracies of the phantom movements were similar to chance (47.5±4.46%, *n*=10). After BMI training, the *F*-value of the contralateral sensorimotor cortex did not increase, although the patient was instructed in the same way as during the first experiment (Fig. 4b). Moreover, the pain scores were not significantly altered, changing from 31.9±15.9 (mean±95% confidence interval) to 32.9±15.9 in VAS (1/100; *n*=10, *P*=0.46, uncorrected, *t*(9)=0.77, paired Student's *t-*test) and from 21.2±19.9 to 23.1±20.8 in total scores of the SF-MPQ2 (*n*=10, *P*=0.063, uncorrected, Wilcoxon signed-rank test). Notably, the order of the two experiments was random (Supplementary Table 3).

As expected, BMI training with the phantom decoder increased the discriminability of the cortical activity representing the phantom hand movements. However, contrary to the naive hypothesis, pain significantly increased after training with the phantom decoder. Therefore, we added a subsequent training experiment with a real hand decoder to reduce the discriminability. The real hand decoder was constructed from MEG signals obtained while moving the intact hand. During training with the real hand decoder, the patients were similarly instructed to freely control the neuroprosthetic hand by trying to move their phantom hand, not the intact hand. Therefore, the patients intended to associate their phantom hand movements with the movements of the prosthetic hand, which was actually controlled by a decoder to classify the MEG signals based on the intact hand's movement. As a result, the patients were expected to unknowingly associate the phantom movements with the cortical representation of the intact hand's movements, which were different from the cortical representation of the phantom movements in pre-BMI training. We expected that BMI training with the real hand decoder would accelerate the dissociation of the link between the phantom hand and the original cortical representation by creating a new link to the real hand. The association of the different neural representation might dissociate the prosthetic hand and the original neural representation of phantom movements even more so than the association of the randomly moved prosthetic hand and the neural representation.

Consistent with this prediction, after BMI training with the real hand decoder, the *F*-values of the phantom hand movements decreased for the sensorimotor cortex contralateral to the phantom hand (Fig. 4c). Moreover, the pain scores decreased significantly from 38.3±15.5 (mean±95% confidence interval) to 34.6±14.8 in VAS (1/100; *n*=10, *P*=0.029, uncorrected, *t*(9)=2.60, paired Student's *t-*test). Similarly, the total scores of the SF-MPQ2 significantly decreased from 26.0±21.0 to 20.7±16.3 (*n*=10, *P*=0.016, uncorrected, Wilcoxon signed-rank test). The *F*-values of the intact hand movements decreased for the sensorimotor cortex contralateral to the intact hand (Supplementary Fig. 4).

To statistically evaluate plastic changes of the discriminability in cortical currents, the differences in the *F-*values before and after training (post–pre) were compared among the training sessions (also see Supplementary Fig. 5 for the alteration in cortical currents). The increases in the *F*-values on the contralateral sensorimotor cortex were significantly larger for the phantom hand decoder than the random decoder (Fig. 4d). In contrast, the decreases in the *F*-values were significantly larger for the real hand decoder than the random decoder (Fig. 4e). Thus, the *F*-values on the contralateral sensorimotor cortex varied significantly after BMI training depending on the decoders.

According to their reports, patients noticed a difference in their ability to control the robotic hand. They attributed the ability to use the robotic hand to themselves; however, they were not aware of the experimental manipulations (Supplementary Table 2). Moreover, no patients reported any subjective feelings of being in control of the prosthetic hand as a part of their body.

### Pain and discriminability of cortical currents

**Figure 5:**
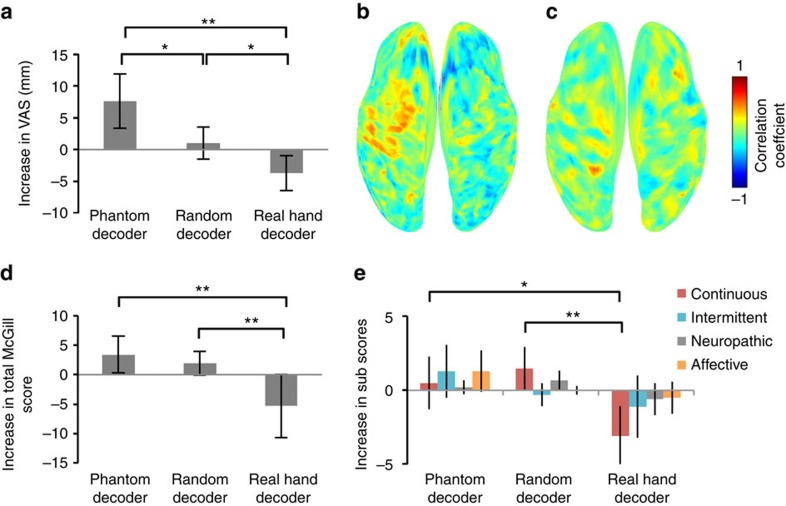
Alteration in pain correlates with alteration in *F*-values for the sensorimotor cortex. (**a**) The averaged differences in VAS scores (post–pre) are shown with the 95% confidence interval for three experiments (*n*=10). The asterisks denote significant differences (*P<0.05, **P<0.01, uncorrected, two-tailed Student's *t-*test). (**b**) The Pearson correlation coefficient between the Δ*F*-value and the ΔVAS at each vertex was colour-coded on the normalized brain (*n*=30). (**c**) The Pearson correlation coefficient between Δcurrent and ΔVAS at each vertex was colour-coded on the normalized brain (*n*=30). (**d**) The averaged differences in total scores of SF-MPQ2 (post–pre) are shown with the 95% confidence interval for three experiments (*n*=10). The asterisks denote significant differences (*n*=10, **P<0.01, uncorrected, Mann–Whitney *U*-test). (**e**) The averaged differences in subscores of the SF-MPQ2 (post–pre) are shown with the 95% confidence interval for three experiments (*n*=10). The asterisks denote significant differences (*n*=10, *P<0.05, **P<0.01, uncorrected, Mann–Whitney *U*-test).

The above analyses showed that the increases in the pain VAS scores were significantly changed depending on the decoder type (*n*=10 each, *P*=0.0002, *F*(2, 27)=11.5, one-way ANOVA; Fig. 5a). After training with the real hand decoder, the VAS scores decreased significantly compared with those of the random decoder and the phantom decoder (*n*=10, *P*=0.025 and 0.0003, uncorrected, *t*(18)=2.45 and 4.36, respectively, two-tailed Student's *t-*test). In contrast, the VAS scores increased significantly after training with the phantom decoder compared with the random decoder (*n*=10, *P*=0.017, uncorrected, *t*(18)=2.62, two-tailed Student's *t-*test). Notably, these increased scores from the phantom decoder spontaneously returned to the previous state after more than 2 weeks and were not significantly different from the scores before the training (*n*=10, *P*=0.55, uncorrected, *t*(9)=0.63, paired Student's *t-*test).

The alteration in the *F*-value was evaluated with respect to pain. For all three experiments, the increase in the *F*-value after BMI training (Δ*F*) was compared with the increase in the VAS scores (ΔVAS). The correlation coefficients between Δ*F* and ΔVAS at each of the vertices were colour-coded on the normalized brain (*n*=30, Pearson correlation coefficient; Fig. 5b). Interestingly, ΔVAS was positively correlated with Δ*F* on the sensorimotor cortex contralateral to the phantom hand. That is, pain increased as the discriminability in cortical currents representing phantom movements increased.

In contrast, the alteration in the mean cortical currents was not significantly correlated with the alteration in pain. The *z-*scored cortical currents were averaged among all trials of grasping and opening for each patient. The increase in the mean cortical currents after BMI training (Δcurrent) was compared with the increase in VAS scores (ΔVAS). No significant correlation was found between ΔVAS and Δcurrent for the sensorimotor cortex (*n*=30, Pearson's correlation coefficient; Fig. 5c, also see Supplementary Fig. 6).

Moreover, the total scores of the SF-MPQ2 varied significantly depending on the decoders (*n*=10, *P*=0.0077, *F*(2, 27)=5.86, one-way ANOVA; Fig. 5d). These scores decreased significantly after training with the real hand decoder compared with the random decoder and phantom decoder (*n*=10, *P*=0.001 and 0.0034, uncorrected, respectively, Mann–Whitney *U*-test). However, the alterations of scores were not significantly different between the phantom decoder and random decoder (*n*=10, *P*=0.59, uncorrected, Mann–Whitney *U*-test). Among the four types of subscores in the SF-MPQ2, only the continuous pain scores varied significantly among the decoders (*n*=10, continuous, *P*=0.0033, *F*(2, 27)=7.12; intermittent, *P*=0.15, *F*(2, 27)=2.07; neuropathic, *P*=0.090, *F*(2, 27)=2.63; affective, *P*=0.064, *F*(2, 27)=3.04, one-way ANOVA; Fig. 5e). This score decreased significantly after training with the real hand decoder compared with the random decoder and phantom decoder (*n*=10, *P*=0.001 and 0.045, uncorrected, respectively, Mann–Whitney *U*-test). The alterations of the continuous scores were not significantly different between the phantom decoder and random decoder (*n*=10, *P*=0.12, uncorrected, Mann–Whitney *U*-test).

### BMI training altered decoding accuracy of phantom movements

**Figure 6:**
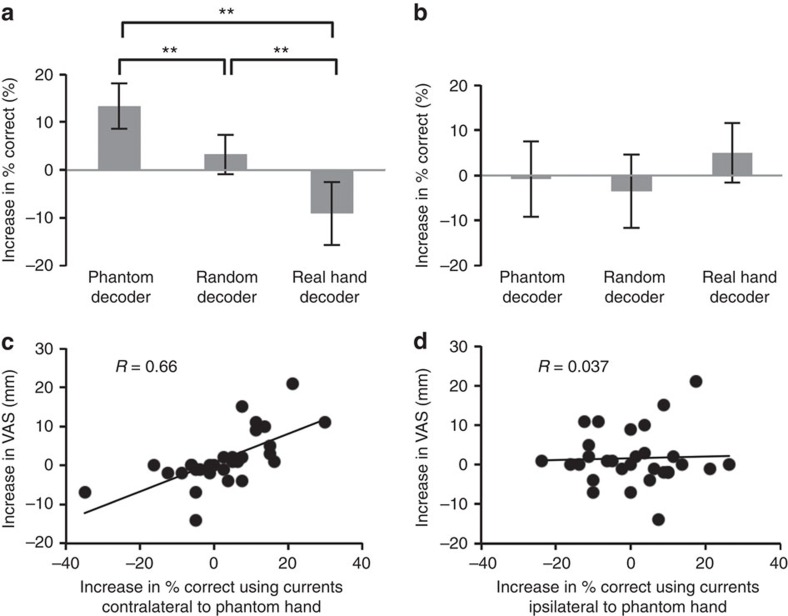
Alterations in classification accuracies among the three experiments were correlated with alterations in pain. (**a**,**b**) The accuracies for classifying two types of phantom movements were evaluated using the currents on the motor cortex contralateral (**a**) and ipsilateral (**b**) to the phantom hand. Each bar shows the average difference in the accuracy with 95% confidence intervals for each experiment. The asterisks denote significant differences (*n*=10, **P<0.01, Bonferroni-corrected, two-tailed Student's *t-*test). (**c**,**d**) The increase in VAS scores was significantly correlated with the increase in the accuracy for classifying the phantom movement types using the *z-*scored cortical currents on the sensorimotor cortex contralateral to the phantom hand (*n*=30; **c**), although the increase in VAS scores was not correlated with that for the cortex ipsilateral to the phantom hand (**d**).

In addition to the univariate analyses, we evaluated the alteration in the cortical currents using the decoding method (multivariate analysis). For all patients, we compared the accuracies for classifying the phantom movements using the estimated currents in the sensorimotor cortex contralateral and ipsilateral to the phantom hand. The differences in accuracies varied significantly among the three conditions using the currents contralateral to the phantom hand (*n*=10 each, *P*=0.00001, *F*(2, 27)=17.52, one-way ANOVA; Fig. 6a). The accuracy decreased significantly after training with the real hand decoder compared with the phantom decoder and random decoder (*n*=10, *P*=0.00004 and 0.006, Bonferroni-corrected, *t*(18)=7.44 and 3.59, respectively, two-tailed Student's *t-*test). Moreover, the classification accuracy increased significantly after training with the phantom decoder compared with the random decoder (*n*=10, *P*=0.006, Bonferroni-corrected, *t*(18)=3.60, two-tailed Student's *t-*test). In contrast, the accuracies using the currents for the sensorimotor cortex ipsilateral to the phantom hand did not significantly change among the three types of decoders (*n*=10 each, *P*=0.25, *F*(2, 27)=1.45, one-way ANOVA; Fig. 6b). In addition, the accuracy in classifying the intact hand movements was not significantly changed after the BMI training with the real hand decoder for the cortical currents of the contralateral or ipsilateral sensorimotor cortex (*n*=10, *P*>0.05 for each, Bonferroni-corrected, two-tailed Student's *t-*test; Supplementary Fig. 4).

### Pain changes with accuracy of classifying phantom movements

The changes in the VAS scores were significantly correlated with the changes in classification accuracy using the currents for the sensorimotor cortex contralateral to the phantom hand (*n*=30, *R*=0.66, *P*=0.0001, Pearson's correlation coefficient; Fig. 6c), but not with the changes in accuracy using the currents ipsilateral to the phantom hand (*n*=30, *R*=0.037, *P*=0.76, Pearson's correlation coefficient; Fig. 6d). In addition, the changes in the total SF-MPQ2 scores were significantly correlated with the changes in accuracy using the contralateral currents (*n*=30, *R*=0.51, *P*=0.0044, Spearman's rank correlation coefficient), but not with accuracy using ipsilateral currents (*n*=30, *R*=−0.14, *P*=0.47, Spearman's rank correlation coefficient).

## Discussion

Our data show that MEG-based BMI training to control a neuroprosthetic hand induces significant changes in pain, and this was directly associated with plasticity of the cortical representation of phantom movements in the contralateral sensorimotor cortex. Specifically, we found that the induced changes in the motor information content (movement discriminability) of the sensorimotor cortex underlaid the effect on the pain. Pain increased significantly in direct proportion to the decoded information content of phantom hand movements; in turn, subsequent training of phantom hand movements based on the intact hand disrupted the information content of the contralateral sensorimotor cortex and led to an improvement in pain. These results are consistent with a probable causal relationship between cortical sensorimotor plasticity and phantom limb pain.

Notably, our results demonstrated that pain was not reduced by the reconstruction of motor function; instead, it was changed by plastic changes in the cortical representation. The reconstruction of motor function does not necessarily reverse a putatively maladaptive cortical representation, which was expected from the naive hypothesis. By selecting the decoded information to control the BMI neuroprosthesis, we induced plasticity in the cortical information representation of phantom hand movements (as opposed to simple topographic plasticity) and explored the relationship with pain^30^.

It is well established that cortical activity representing phantom hand movements is preserved in the sensorimotor cortex^14^,^16^. However, it has remained unclear whether and how pain might depend on altered representations within this region^11^. Most previous studies examined the topography of sensorimotor function, for instance, showing that cortical representations of phantom hand movements overlap the adjacent cortical representation of mouth movements^7^, with a larger overlap corresponding to greater pain. However, recent data have contradicted this^13^ and are not completely consistent with other evidence showing that greater pain is associated with higher cortical activation representing phantom hand movements^14^,^31^. These seemingly conflicting results have left open the question of whether and how phantom pain might depend on the cortical plasticity of phantom hand representations.

Our approach provides an interventional method to induce localized changes in cortical representations and to directly and reversibly study the relationship with pain. The MEG signals during the offline task were decoded with accuracy comparable to that of previous work, demonstrating the successful online control of a robotic hand with accuracy greater than that of chance^23^. BMI training with the robotic hand was shown to induce robust changes in cortical activity and pain according to the type of the decoder. Cortical plasticity was demonstrated by two different analysis approaches. Univariate analysis showed that alterations in pain were significantly correlated with alterations in discriminability of cortical currents between movement types, as opposed to the mean (absolute) cortical current magnitude. Multivariate analysis based on motor decoding showed clear modulation of information content pertaining to phantom hand movements, which was shown to be strongly correlated with the modulation of pain. Notably, the proportional relations were significant both with VAS and the total SF-MPQ2 scores. Although the increases in total SF-MPQ2 scores were not significant after training with the phantom decoder, the VAS presumably more sensitively captured moment-by-moment pain than the SF-MPQ2 did. Thus, it is likely that it is primarily the functional information content of cortical phantom limb representations that is causally related to pain: pain increases with the enhancement of information about phantom hand movements.

As the patients were unaware of the experimental manipulation in each case, it is unlikely that placebo effects were dominant. Other potential aspects of BMI neuroprosthetic control, such as a nonspecific training effect or emotional pain modulation by perceived task difficulty^9^,^32^, are not likely confounders because these were broadly equivalent across task conditions (Supplementary Table 2). In addition, although mismatches between expected and actual movements of the robotic hand were different among task conditions, the mismatches did not explain the alterations among the task conditions. The random decoder should be the most difficult and cause the largest mismatches; however, this did not change pain. Therefore, our results strongly suggest that the induced plasticity on the phantom limb representation could be the cause of the alteration in pain. It should be noted, however, that the training with the real hand decoder was performed as the last experiment. One limitation of this study is that the order of three experiments was not counterbalanced.

The estimation of cortical currents from the MEG signals and the decoding method applied to the estimated cortical currents revealed that the information about hand movements was evaluated differentially in both hemispheres. The information of the phantom hand was significantly lateralized to the sensorimotor cortex contralateral to the affected hand. In addition, the alteration of information due to the BMI training was significant only in the contralateral hemisphere. Moreover, for the same contralateral hemisphere, the information of the hand movements was significantly different among the phantom hand and intact hand. The information of intact hand movement was not significantly different after training with the real hand decoder. These results strongly suggest that the BMI training in this study altered the information of the phantom hand movement in the contralateral hemisphere significantly enough to change the pain. However, a recent study suggested the that ipsilateral hemisphere may affect the pain^33^. In addition, the real hand decoder might affect the contralateral hemisphere through some plastic changes in the ipsilateral hemisphere. Thus, further investigation is necessary to reveal the relationship between both hemispheres to control the pain.

Researchers have proposed an incongruence hypothesis of pain^34^ based on prediction errors between actual and predicted somatosensory feedback estimated by an internal model in the brain^35^,^36^,^37^. A mismatch between the actual experience and the predicted experience might be a causative component of chronic pain^38^. This hypothesis was proposed as a mechanism for the noted success of mirror therapy to reduce pain (that is, by restoring consistency between motor intention and visual input). In our experiment, BMI training with a phantom decoder might have increased pain by enhancing the cortical representation that was associated with the internal model generating inconsistency in sensorimotor functions. However, BMI training with the real hand decoder might have dissociated the intention to move the phantom hand and the cortical representation, resulting in deteriorated information of phantom representation and reducing pain.

Our results show that BMI training may be useful as a novel interventional tool to study functional anatomy in the brain. It induces functionally specific plastic changes in the targeted cortex based on the information represented in the activity. Moreover, this method can be easily applied to patients in non-invasive, randomized and blinded studies. These features distinguish this method from other non-invasive interventional tools, such as transcranial direct current stimulation^39^ and transcranial magnetic stimulation^40^. Although these modalities change cortical activity and excitability to relieve pain^41^, it is difficult to control the information represented in the patterns of cortical activity. Therefore, if the information of the phantom hand is the cause of the phantom limb pain, then the simple application of the transcranial direct current stimulation and transcranial magnetic stimulation might not be enough for full recovery. However, cortical stimulations have been demonstrated to modulate cortical activities to relieve pain^41^. With this in mind, it is potentially interesting to combine cortical stimulation and online neural decoding^42^. The novel neurofeedback of cortical stimulation based on the neural information might reveal the cause of phantom limb pain and allow a full recovery.

BMI neurofeedback might be a potential novel therapy for phantom limb pain. A previous study showed that the effective rate of mirror therapy was limited^43^. Five patients in this study had some experience with mirror therapy (Table 1), but the treatment was effective for only one patient during the limited period. However, we observed that appropriate BMI training reduced pain even in these patients (Supplementary Table 3), suggesting that it could be an alternative to mirror therapy. Moreover, some patients in this study had also undergone lesioning of the dorsal root entry zone (DREZotomy)^44^ and reported residual pain after surgery. The BMI training significantly reduced the scores for continuous pain in the residual pain. By combining decoding^45^ and neurofeedback, BMI could be applied to other chronic pain conditions.

In addition, the results clearly indicate that we should consider pain as a potential complication when using BMIs for paralysed patients. That is, we must consider the fact that true decoding that increases pain creates a problem for those patients using BMI-controlled robotic prostheses. One possible solution, according to the incongruence hypothesis, would be BMI training accompanied by sensory feedback (for example, artificial–real nerve coupling^46^), which might mitigate pain by providing an intact sensorimotor loop.

In summary, neurofeedback training using MEG-based BMI provides a novel method to directly change the information content of motor representations by induced plasticity in the sensorimotor cortex. Here we showed that BMI training to enhance phantom limb representation was associated with increased pain, and that BMI training to deteriorate the representation reduced pain. This suggests a direct and causative link between sensorimotor cortical plasticity and pain in phantom limb patients, and that BMI training may be a novel and clinically useful treatment.

## Methods

### Subjects

Nine brachial plexus root avulsion (BPRA) patients and one amputee (all males; mean age, 51.7 years; range, 38–60 years) all of whom had pain in their phantom limb participated in this study (Table 1 and Supplementary Table 1). We had 12 patients with pain in their phantom limb at the Department of Neurosurgery at Osaka University Hospital from January 2012 to July 2015. Among these patients, we selected 10 who met all of the following inclusion criteria: (1) pain and phantom sensation in the upper limb; (2) no hand or no sensation in the residual hand; (3) severe paresis with manual muscle testing score 0–1; and (4) normal comprehension and intellectual capacity according to the Japanese Adult Reading Test25. The total number of patients was chosen to be comparable to that of previous studies and was based on our preliminary results for healthy controls trained by the same BMI prosthesis^6^,^10^. Notably, 9 of the 10 were the same patients who also took part in our previous study^23^. The study adhered to the Declaration of Helsinki and was performed in accordance with protocols approved by the Ethics Committee of Osaka University Clinical Trial Center (no. 12107, UMIN000010180). All patients were informed of the purpose and possible consequences of this study, and written informed consent was obtained. In figures, the photo of the patient was used with the patient's permission for publication.

Our patient group included one amputee and nine BPRA patients with complete avulsion of roots from C5 to Th2. According to Flor *et al*.^3^, phantom limb pain belongs to a group of neuropathic pain syndromes that is characterized by pain in the amputated limb or pain that follows partial or complete deafferentation. Although the amputation and the BPRA were largely different regarding the existence of the residual arms, we included them as phantom limb pain when the patient met all the aforementioned criteria. Notably, the affected hands of all the BPRA patients had no sensation and were plegic because of the complete avulsion of their roots (as definitively confirmed through magnetic resonance imaging (MRI) and computed tomography with myelogram). Although some of them were able to slightly contract the muscles in their upper arms because of intercostal nerve transplantation, they were not aware of the contraction without observing the arm. Moreover, without observing the physical hand, they complained of the existence of their hand, which they felt was slightly movable in their mind, and suffered from intractable pain in the insensible hand. These properties are the same as those described for the phantom limb due to amputation. However, it should be also noted that the pain after BPRA may not be precisely the same as the phantom limb pain after amputation. Pain after BPRA is caused by several factors, as is the pain after amputation^3^. In particular, the paroxysmal pain after BPRA is hypothesized to originate from the affected spinal cord and is often relieved by DREZotomy^44^. Moreover, in BPRA patients, some motor and sensory functions of the arm partially remain. Therefore, not all pain after BPRA are necessarily phantom limb pain. We hypothesized that a part of the pain in the insensible and plegic hand of BPRA patient originated from the same mechanism causing a part of the pain in the amputee, which was affected by the cortical plasticity in the sensorimotor cortex representing the phantom hand movements. To characterize the properties of the pain, we analysed the subscores of SF-MPQ2.

### MEG recording

For the MEG recording, subjects were in the supine position with the head centred in the gantry. A projection screen in front of the face presented visual stimuli using a visual stimulus presentation system (Presentation; Neurobehavioral Systems, Albany, CA, USA) and a liquid crystal projector (LVP-HC6800; Mitsubishi Electric, Tokyo, Japan; Fig. 1a). MEG signals were measured by a 160-channel whole-head MEG equipped with coaxial-type gradiometers housed in a magnetically shielded room (MEGvision NEO; Yokogawa Electric Corporation, Kanazawa, Japan).

The MEG signals were sampled at 1,000 Hz with an online low-pass filter at 200 Hz and acquired online by FPGA DAQ boards (PXI-7854R; National Instruments, Austin, TX, USA) after passing through an optical isolation circuit. The signals for the 84 selected channels were used for offline analysis and online control of the prosthesis. Subjects were instructed not to move their head to avoid motion artefacts. A cushion was placed under the intact elbows to reduce motion artefacts.

Five head marker coils were attached to the subject's face before beginning the MEG recording to provide the position and orientation of MEG sensors relative to the head. The positions of the five marker coils were measured to evaluate the differences in the head position before and after each MEG recording. The maximum acceptable difference was 5 mm.

We also recorded electromyograms of the face and forearm to monitor muscle activities (Supplementary Fig. 1). Subjects were monitored by two video cameras to confirm their arousal.

### Experimental design

All patients participated in a crossover trial consisting of three experiments on different days. Each experiment consisted of three tasks: offline task (pre-BMI), BMI training and offline task (post-BMI; Fig. 1b). First, in the offline task, the patients attempted to move their phantom hands or their intact hands (grasping and opening) at the presented times^16^ while the MEG signals of selected channels were recorded. The acquired MEG signals were used to construct the decoder to control the robotic hand. Then, the subjects were instructed to control the prosthetic hand in real time using the trained decoder.

The experiment was performed three times with different decoders. Each experiment was performed after more than 2 weeks had passed since the previous experiment. For the experiments with the phantom decoder and random decoder, the order of the experiments was randomly assigned to the patients to balance group sizes. The experimenter was not blinded to the group allocation. After two experiments, the experiment with the real hand decoder was performed; that is, the real hand decoder experiment was always performed last. At first, we designed the experiments to compare the phantom decoder and random decoder according to the naive hypothesis. The third experiment with the real hand decoder was added to decrease the pain. We selected this study design for ethical considerations of not increasing the patient's pain.

At the time of enrolment in this trial, we instructed the patients to use their brain activity to control the robotic hand; however, they were not informed of changes in the decoders throughout the experiments.

### Offline task

In the offline task, the patients were instructed to attempt to move their phantom hand or to move their intact hand (Fig. 2b). The patients were visually instructed regarding the movement type to perform with the Japanese word for ‘grasp' or ‘open.' After the movement-type instruction, four execution cues were given to the subject every 5.5 s. The execution cue was given both visually and aurally, and was presented 40 times for each movement type. The order of the requested movement type was random. Before the phantom hand task, we instructed the patients to attempt to slightly move the phantom hand once at the cued time without moving other parts of their bodies. It should be noted that the attempt to move the phantom hand is different from the motor imagery of the phantom hand^47^,^48^. Before the first experiment, we explained this difference and explicitly instructed them to attempt to move the phantom hands. For the intact hand task, we instructed the patients to slightly move the intact hand once at the cued time, without moving other parts of their bodies.

The subjective confidence in moving the phantom hand was different among patients (Supplementary Table 1). Although the speed with which the phantom hand was moved differs among patients^49^, all patients in this study were able to grasp and open the phantom hand slightly within 2.3 s, which was sufficient to complete the instructed task before the cue for the next trial.

### BMI training

During BMI training, a monitor in front of each subject showed a picture of the prosthetic hand in real time for visual feedback (Fig. 1a). Patients were instructed to control the prosthetic hand freely for 10 min to improve their ability to control it by intending to grasp or open the phantom hand. At the beginning of the training, the experimenter changed the threshold to detect the onset. Because the optimal threshold estimated from the offline task was sometimes lower than the estimated values of the onset detector during resting in the online task, we changed the threshold values to not detect the onset during the resting state in the online task, although the other parameters estimated from the offline task were not changed^23^. The selected parameters were fixed for the 10 min of training.

### Decoder to control the prosthetic hand

MATLAB R2013a (Mathworks, Natwick, MA, USA) was adopted to calculate the decoding parameters and for online prosthetic hand control. First, MEG signals from the 84 selected sensors in the offline session were averaged in a 500-ms time window and converted to the *z*-score using the mean and s.d. estimated from the initial 50 s of data during the offline session. The time-averaged MEG signals were calculated for the period from −2,000 to 1,000 ms, at 100-ms intervals according to the execution cue.

The *z-*scored signals in the offline session were used to train the online decoder, which consisted of an onset detector and class decoder to control the prosthetic hand online in the following BMI training session. The class decoder was trained at the peak classification accuracy of the offline task. The onset detector was trained using the time-averaged signals of 500-ms windows slid by 100 ms from −500 to 1,000 ms, with respect to the timing of the instruction to move. The details of the construction of the decoder are available in our previous report^20^.

Here we constructed three types of online decoders depending on the data used to train the decoder. The phantom decoder was trained by the MEG signals of the offline task to move the phantom hand. The random decoder was trained by the MEG signals of the same offline task with randomized types of movements. The real hand decoder was trained by the MEG signals of the offline task to move the intact hand.

### Classification of movement types in the offline task

Classification accuracy of the movement type was estimated by 10-fold nested cross-validation, which was adopted so that hyperparameters for the SVM and time window were always selected independently from the testing data set (also see our previous report^20^). To optimize the hyperparameters and the time window, training data sets were classified by 10-fold cross-validation 10 times, and the parameters with the highest average classification accuracy of the repeated cross-validations were selected. The classification accuracy was calculated from the classification result of each testing data set, which was tested by the decoder trained with the optimized hyperparameters and time window. All decoding analyses were performed with MATLAB R2013a using radial basis function kernel SVM.

### Cortical current estimation by VBMEG

A polygonal model of the cortical surface was constructed based on structural MRI (T1-weighted; Signa HDxt Excite 3.0 T; GE Healthcare UK Ltd, Buckinghamshire, UK) using the Freesurfer software (Martinos Center Software)^50^. To align MEG data with individual MRI data, we scanned the three-dimensional facial surface and 50 points on the scalp of each participant (FastSCAN Cobra; Polhemus, Colchester, VT, USA). Three-dimensional facial surface data were superimposed on the anatomical facial surface provided by the MRI data. The positions of five marker coils before each recording were used to estimate cortical current with VBMEG.

VBMEG is a free software for estimating cortical currents from MEG data (ATR Neural Information Analysis Laboratories, Kyoto, Japan)^51^,^52^. VBMEG estimated 4,004 single-current dipoles that were equidistantly distributed on and were perpendicular to the cortical surface. An inverse filter was calculated to estimate the cortical current of each dipole from the selected MEG sensor signals. The hyperparameters m0 and γ0 were set to 100 and 10, respectively. The inverse filter was estimated by using MEG signals in all trials from 0 to 1 s in the offline task, with the baseline of the current variance estimated from the signals from −1.5 to −0.5 s. The filter was then applied to sensor signals in each trial to calculate cortical currents.

### Code availability

The code used in this study is available by contacting the corresponding author (T.Y.).

### Data availability

The data that support the findings of this study are available on request from the corresponding author (T.Y.). The data are not publicly available because they contain information that could compromise research participants' privacy and/or consent.

## Additional information

**How to cite this article**: Yanagisawa, T. *et al*. Induced sensorimotor brain plasticity controls pain in phantom limb patients. *Nat. Commun.* **7**, 13209 doi: 10.1038/ncomms13209 (2016).

## Supplementary Material

Supplementary Information Supplementary Figures 1 – 6 and Supplementary Tables 1 – 3

Supplementary Movie 1 This movie shows how the patient controlled the prosthetic hand during the BMI training. The patient freely controlled the prosthetic hand to grasp and release a ball by intending to move his phantom hand while watching the movements of the prosthetic hand.

## Figures and Tables

**Table 1:** Clinical profiles of patients.

**Patient ID**	**Age (y)/sex**	**Diagnosis**	**JART FSIQ/VIQ/PIQ**	**Disease duration (y)**	**Mirror therapy**
1	50/M	Right BPRA of C6–8	100/100/90	34	Effective only for a short period
2	51/M	Left BPRA of C5–Th1	96/96/96	6	Not effective
3	58/M	Right BPRA of C6–Th2	112/114/108	40	No experience
4	48/M	Amputation below right elbow	104/105/102	1.5	Not effective
5	49/M	Right BPRA of C7–Th1	102/102/101	29	No experience
6	56/M	Right BPRA of C7–8	114/116/110	38	Not effective
7	51/M	Right BPRA of C6–Th1	110/112/107	11	No experience
8	56/M	Left BPRA of C7–Th1	83/82/87	13	Not effective
9	38/M	Right BPRA of C6–8	85/84/89	21	No experience
10	60/M	Right BPRA of C6–8	114/116/110	20	No experience

BPRA, brachial plexus root avulsion; FSIQ: full-scale intelligence quotient; JART, Japanese Adult Reading Test; M, male; PIQ, performance intelligence quotient; VIQ, verbal intelligence quotient; y, year.
