# Supplementary material for: Induced sensorimotor brain plasticity controls pain in phantom limb patients
Source: Nat Commun. 2016 Oct 27;7:13209. doi: 10.1038/ncomms13209 (PMC5095287; doi:10.1038/ncomms13209)
Supplement: Supplementary Information — Supplementary Figures 1 – 6 and Supplementary Tables 1 – 3 [file ncomms13209-s1.pdf]

Supplementary Figures

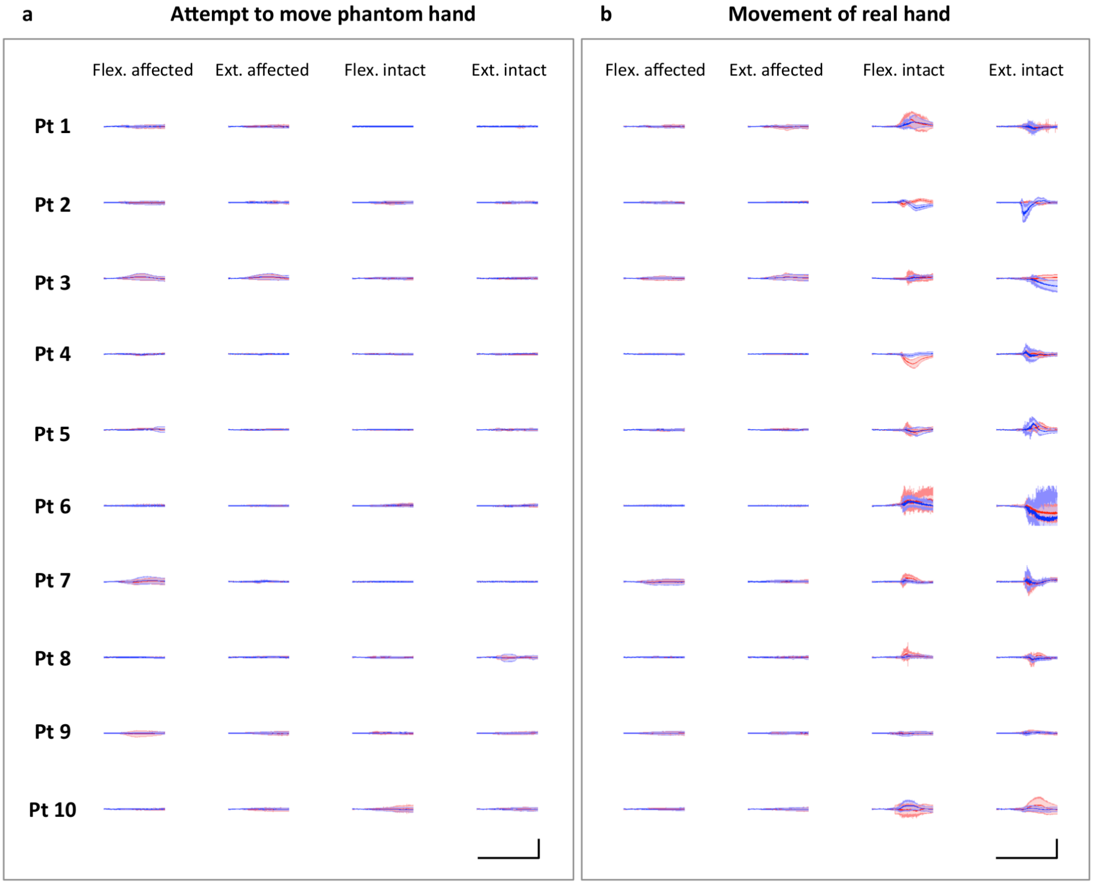

Supplementary Figure 1. EMG response during the offline tasks.

The EMG responses were averaged according to the movement cue (time 0) among grasping (red) and opening (blue) of the phantom hand **(a)** and the real hand **(b)**. The *z*-scored EMG normalized by the signals from –2 s to –1 s is shown for each patient. The EMG was recorded from the flexor digitorum superficialis (Flex.) and the extensor digitorum communis (Ext.) of each hand. For the amputee (Pt 4), the EMG electrodes were placed on the medial and external side of his stump. The length of vertical scale bar is 50. The horizontal bar represents the time from −2 s to 2 s.

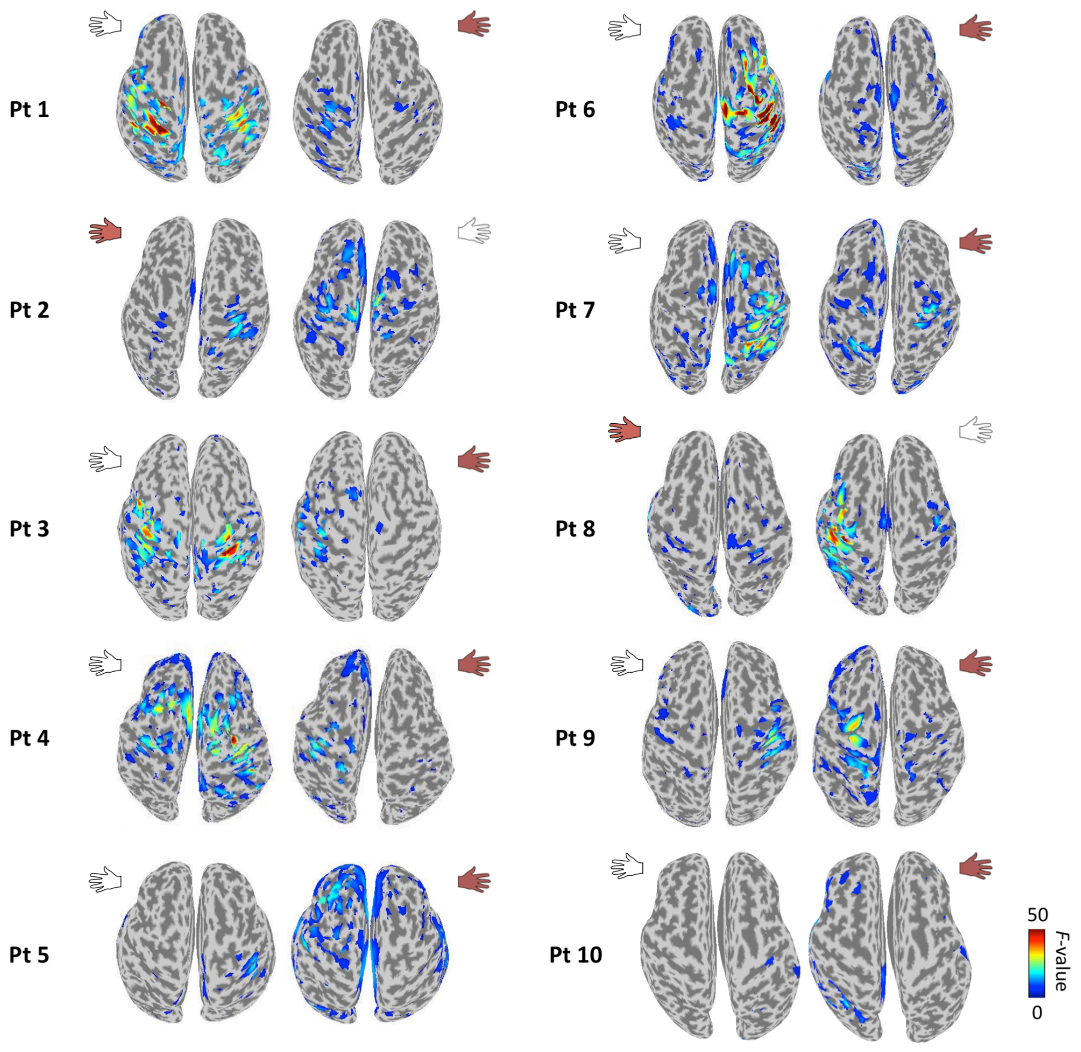

Supplementary Figure 2. *F*-values of the estimated cortical currents of all patients.

For all patients, the *F*-values of ANOVA between the cortical currents during grasping and opening of the real hand and phantom hand were color-coded on the reconstructed brain surface. Only the significant *F*-values are shown ($p < 0.05$, one-way ANOVA). The *F*-values were calculated using the *z*-scored cortical currents at the time of movement onset. The red hand shows the side of the phantom hand.

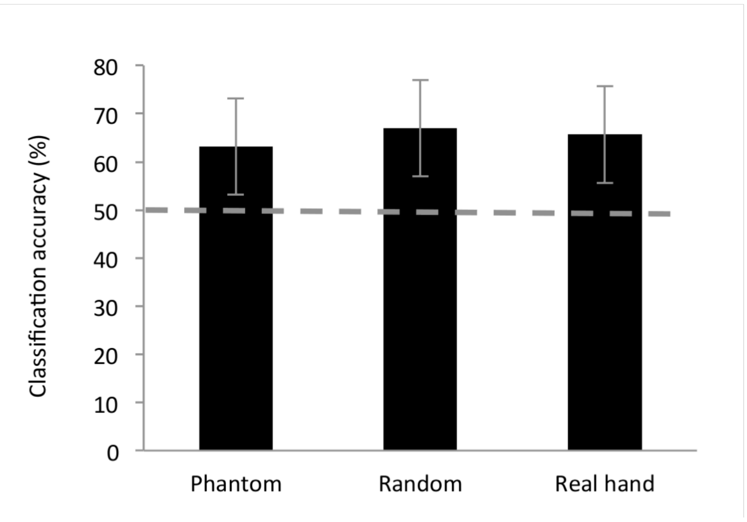

Supplementary Figure 3. Classification accuracies of the sensor-based feature.

The classification accuracies of phantom movements before BMI training (pre-BMI) were not significantly different among the three experiments (n = 10). The bars indicate the mean values, and the error bars show the 95% confidence intervals.

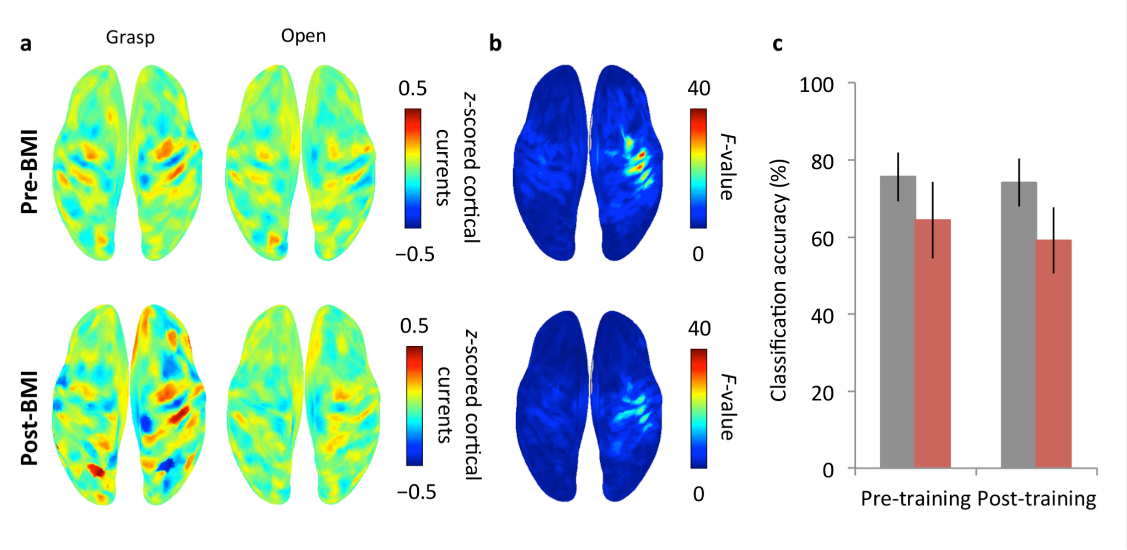

Supplementary Figure 4. The cortical representation of the intact hand during the training with the real hand decoder

(a) For the training with the real hand decoder, the mean *z*-scored cortical currents during grasping and opening of the real hand were color-coded on a normalized brain surface at the time of movement onset (the side of the real hand is shown on the left, $n = 10$). **(b)** The mean *F*-values of ANOVA of the *z*-scored cortical currents between the two movements were color-coded on the normalized brain surface (the side of the real hand is shown on the left, $n = 10$). **(c)** The accuracy of classifying the two movements of the real hands was evaluated using two different features for pre- and post-trainings. The averaged accuracies are shown with a 95% confidence interval (error bar) for each feature ($n = 10$). The accuracies were not statistically different between pre- and post-trainings for each hemisphere ($p > 0.05$ for each, uncorrected, $n = 10$, Student paired *t*-test). Gray: the accuracy using the estimated cortical currents on the sensorimotor cortex contralateral to the intact hand; red: that using the estimated cortical currents on the sensorimotor cortex ipsilateral to the intact hand.

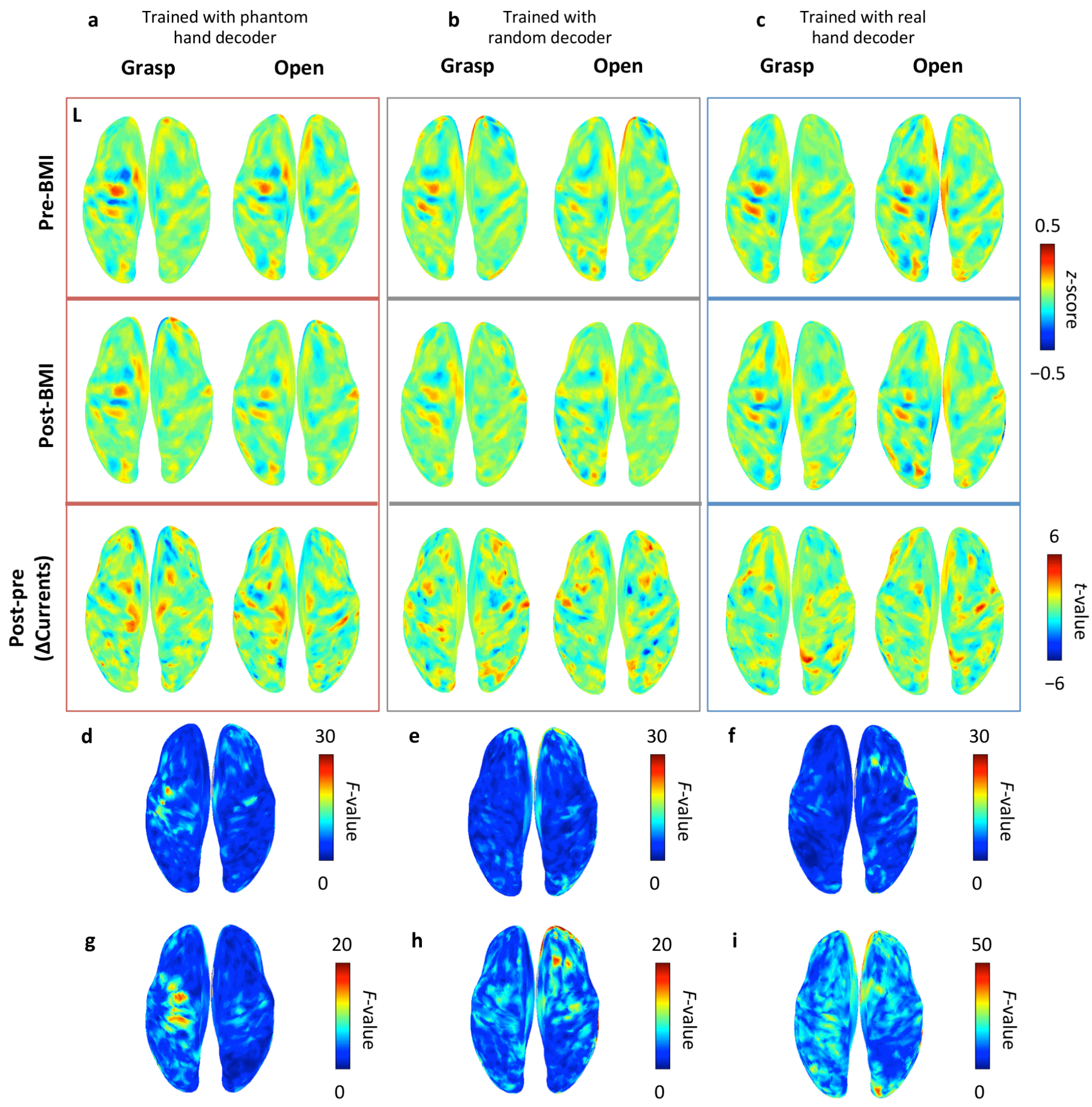

Supplementary Figure 5. Cortical currents vary significantly among the experimental conditions.

The *z*-scored cortical currents during grasping or opening of the phantom hand were averaged at each vertex for the 10 patients and color-coded on the normalized brain surfaces for pre-BMI (upper panel) and post-BMI (middle panel). Each box corresponds to each experiment (**a**, phantom hand decoder; **b**, random decoder; **c**, real hand decoder). The *t*-value of the paired Student *t*-test between the *z*-scored cortical currents of post-BMI and pre-BMI was color-coded on the normalized brain surface (lower panel). **(d–f)** The *F*-values of two-way ANOVA for the

factor of pre vs. post (two movements × pre- and post-BMI) were color-coded on the normalized brain surface for each training. **(g–i)** The *F*-values of three-way ANOVA for each factor. **(g)** grasp vs. open; **(h)** pre- vs. post-BMI; **(i)** three decoders.

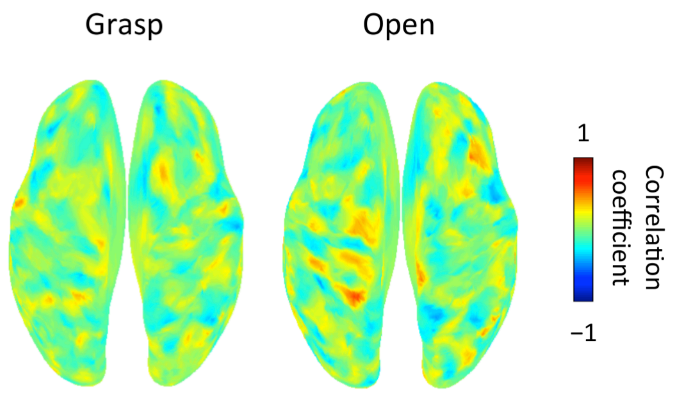

Supplementary Figure 6. Correlation coefficient between the alteration in the cortical currents and the increase in pain.

The Pearson's correlation coefficient between the Δcurrents and the ΔVAS at each vertex was color-coded on the normalized brain for grasping and opening (n = 30).

Supplementary Tables

Supplementary Table 1. Phantom sensation of each patient

ID	Phantom sensation
Pt 1	Continuous pain from fingertips to the forearm of his phantom limb. In particular, severe pain at the fingertips, finger joints, and wrist. Continuous pain is as if the hand was wedged in a door. After coagulation of dorsal root entry zone (DREZotomy), his shooting pain almost disappeared. However, some continuous pain remains. He could slightly move his phantom hand to grasp and open. It took 21.8 s to grasp and open his phantom hand 10 times within the range he could move the hand.
Pt 2	Phantom limb shrinks to the middle of his left forearm. Pain in the thumb to the third finger of his phantom hand. The pain becomes worse when it is cold. Sometimes, he has shooting pain in his phantom hand even after DREZotomy. He could slightly move the fingertips of his phantom hand. It took 18.9 s to grasp and open his phantom hand slightly 10 times within the range he could move the hand.
Pt 3	He had pain consisting of pins-and-needles sensations, such as burning on his elbow and tip of his hand. The pain continues at a numerical rating scale (NRS) of 3–4. The pain was strong especially when it was cold. Sometime, he woke up due to pain at night. He had no shooting pain after DREZotomy. He could slightly move the fingertips of his phantom hand. It took 22.5 s to grasp and open his phantom hand slightly 10 times within the range he could move the hand.
Pt 4	His right hand was amputated due to an accident during his work, for which he was controlling a milling machine. Phantom hand is located inside the stump. Previously, the phantom hand was located outside the stump without a forearm. The hand shrank gradually and was embedded in the stump. Usually, his phantom hand stays in a slightly opened posture. He felt his hand was embedded in cement, and it felt like leaden pain. He

also has sporadic pain. DREZotomy was not performed. He could move each finger of his phantom hand slightly. It took 5.4 s to grasp and open his phantom hand slightly 10 times within the range he could move the hand.

Pt 5 He felt pain around his fifth finger as if it was being screwed by a vise. The pain lasted for a short time and recurred many times a day. The pain still remains after DREZotomy. Pins-and-needles pain always exists in his right hand. He could move his phantom hand almost similar to the intact hand. It took 16.4 s to grasp and open his phantom hand 10 times within the range he could move the hand.

Pt 6 He has continuous pins-and-needles pain and paroxysmal shooting pain in his right hand. His phantom hand always exists in a slightly opened posture and feels as if it is embedded in sand. The feeling of the pain is similar to the feeling when thousands of ants crawl on his hand. He has not yet undergone DREZotomy. He could slightly move the fingertip of his phantom hand. It took 15.8 s to grasp and open his phantom hand 10 times within the range he could move the hand.

Pt 7 He felt pins-and-needles and throbbing pain in his right forearm that was especially strong on the tip of his phantom hand. He also has paroxysmal pain that feels as if the limb is frozen and constricted, with electricity shooting. He has not yet undergone DREZotomy. He could slightly move each finger of his phantom hand. It took 19.8 s to grasp and open his phantom hand 10 times within the range he could move the hand.

Pt 8 He feels pins-and-needles pain in his left hand. Before DREZotomy, he had paroxysmal shooting pain as if his hand was being bitten by several dogs. He could slightly move his phantom hand, but not individual fingers. It took 15.9 s to grasp and open his phantom hand 10 times within the range he could move the hand.

Pt 9 His phantom hand sensation started 2 months after a traffic accident. He has a phantom sensation and pain in the right hand. The phantom hand is located mostly at the same

	position of the real hand but is sometimes misaligned from the wrist. He feels a pins-and-needles sensation of pain. He feels a strong pain as if his forearm is being cinched tight twice by a pincher for 1 minute. He underwent a nerve block treatment. He did not undergo mirror therapy or DREZotomy. His phantom hand stays in a slightly opened posture and hardly moves intentionally. It took 15.6 s to grasp and open his phantom hand 10 times within the range he could move the hand.
Pt 10	He has a phantom sensation and pain in his right hand and forearm. Moving his phantom hand was difficult for him, but he could move the each finger. He feels a pins-and-needles sensation of pain in his right hand with a NRS of 2–3/10 on all days. He has paroxysmal shooting pain with a NRS of 6–7 in his right hand 5 or 6 times a day. He did not undergo mirror therapy or DREZotomy. It took 9.8 s to grasp and open his phantom hand 10 times within the range he could move the hand.

Supplementary Table 2. Subjective reports after training

ID	Task	Comments of patients after training
Pt 1	Phantom	I could control the prosthetic hand for grasping, but it was difficult to control it for opening. I was able to better control it in the later part of the training. The pain increased during the training.
	Random	I became a little better able to control it in the later part of the training. The prosthetic hand was closed when I grasped the phantom hand slowly from the thumb to the fifth finger. Also, the prosthetic hand opened when I opened the phantom hand slowly from the fifth finger to the third finger.
	Real	I could easily control the prosthetic hand by grasping my phantom hand strongly. It seemed that the strong grasping decreased the time delay between my intention to move and the movement of the prosthetic hand. On the other hand, a slight movement of the phantom hand resulted in a longer time delay. I felt tired because I made a strong effort to grasp and open, although my pain decreased because I felt that I was moving my own right hand. In the previous two cases, I just controlled the prosthetic hand, not my own hand.
Pt 2	Phantom	I feel I became able to control the prosthetic hand. I felt tired.
	Random	I could not move the prosthetic hand well.
	Real	I became better able to control the prosthetic hand in the later part of the training. However, the arm sometimes moved when I did not intend to move the arm. I thought that controlling my breath improved the accuracy of controlling the prosthetic hand. I breathed in during grasping and out during opening.
Pt 3	Phantom	I felt I became better able to control the prosthetic hand. I was tired.
	Random	I could not control the prosthetic hand well. The prosthetic hand did not move as I

		intended.
	Real	It was difficult for me to control the prosthetic hand for a grasping posture. But it was rather easy to control it for an opening posture. I felt I could learn to control the prosthetic hand.
Pt 4	Phantom	I could control the prosthetic hand for opening at almost the same timing when I intended to open my phantom hand. However, it was difficult to control the hand for grasping. My feeling of numbness in the phantom hand increased after the training. Also, I felt something like muscle aches in my phantom hand. The pain seems to be concentrated on the tip of the phantom hand after the training. Usually, the pain was distributed uniformly from the elbow to hand. However, after the training, the pain was increased in the hand and decreased around the elbow.
	Random	I could not control the prosthetic hand well. The prosthetic hand did not move according to the intended timing.
	Real	I could not master the techniques to control the prosthetic hand. I could easily control it for grasping, but opening was difficult. The prosthetic hand opened at a time when I did not intend it to open. Also, I could not sustain the opening posture.
Pt 5	Phantom	It was difficult to control. I felt a little improvement in control after the training, although it was difficult to control both grasping and opening.
	Random	I could control the prosthetic hand for grasping, but it was difficult to control it for opening.
	Real	I could control the prosthetic hand for grasping, but it was difficult to control it for opening. The pain improved slightly, perhaps because I concentrated on the task.
Pt 6	Phantom	I felt I could control the prosthetic hand better than the previous case (Random). The prosthetic hand moved at the intended time.
	Random	The prosthetic hand opened at an unintended time. I could not maintain grasping with the

		prosthetic hand. I felt that controlling it was difficult, because I have not thought about moving my phantom hand for a long time.
	Real	It was difficult to control the prosthetic hand, although I tried various ways to move my phantom hand. During the middle of the training, I felt that I could control the prosthetic hand. But at the end of the training, the prosthetic hand seemed to move against my intentions.
Pt 7	Phantom	I could control the prosthetic hand better than I expected. The accuracy for controlling the hand improved in the later part of the training. I felt strong pain attacks several times during the training.
	Random	It was difficult to control the prosthetic hand. Opening of the prosthetic hand was controlled by imagining hand postures of coded signs during a basketball game. I did not feel a significant change in pain, although I felt a small increase in pain after the training.
	Real	The prosthetic hand was successfully grasped by imaging a grasping posture of the right phantom hand with my eyes closed. Opening the prosthetic hand was difficult. The accuracy for controlling the prosthetic hand improved in the later part of the training.
Pt 8	Phantom	Controlling the prosthetic hand was difficult. In my count, the accuracy for following my intention was about 50%, although this improved in the later part of the training. The prosthetic hand opened when I did not intend to open my phantom hand. During the training, I felt some numbness on the palm of my phantom hand.
	Random	The prosthetic hand did not move according to my intention. The pain did not change much.
	Real	Controlling the prosthetic hand was difficult. I did not feel any changes throughout the training.
Pt 9	Phantom	Controlling the prosthetic hand was difficult. I think I learned to perform grasping, although opening the prosthetic hand was difficult. I felt that my pain increased during the

		training, although it decreased after the training.
	Random	I could control the prosthetic hand for a grasping posture with almost 100% accuracy. Opening was more difficult. The prosthetic hand opened when I did not intend it to move. My pain increased a little.
	Real	I learned to control the prosthetic hand well compared to the previous experiment [random]. I could sometimes open the prosthetic hand when I intended. Opening was still difficult compared to grasping.
Pt 10	Phantom	Controlling the prosthetic hand was difficult. I could not improve my ability to control the prosthetic hand. I felt that my pain was unchanged.
	Random	Controlling the prosthetic hand was difficult. I think I was able to improve my ability to control the prosthetic hand a little in the later part of the training. My pain was unchanged.
	Real	Controlling the prosthetic hand was difficult. I developed no change in my ability to control the prosthetic hand. My pain was unchanged.

Supplementary Table 3. Order of experiments

ID		1st day	2nd day	3rd day
Pt 1	Experiment	Random	Phantom	Real
	VAS (Pre/Post)	6 / 2	32 / 43	27 / 13
	SF-MPQ2 (Pre/Post)	4 / 4	6 / 7	6 / 5
Pt 2	Experiment	Phantom	Random	Real
	VAS (Pre/Post)	0 / 2	1 / 1	4 / 4
	SF-MPQ2 (Pre/Post)	0 / 1	1 / 1	1 / 1
Pt 3	Experiment	Phantom	Random	Real
	VAS (Pre/Post)	10 / 25	22 / 25	26 / 26
	SF-MPQ2 (Pre/Post)	3 / 9	4 / 4	9 / 6
Pt 4	Experiment	Phantom	Random	Real
	VAS (Pre/Post)	52 / 57	48 / 48	42 / 40
	SF-MPQ2 (Pre/Post)	5 / 6	5 / 5	4 / 4
Pt 5	Experiment	Random	Phantom	Real
	VAS (Pre/Post)	59 / 58	54 / 55	61 / 54
	SF-MPQ2 (Pre/Post)	95 / 96	28 / 27	46 / 27
Pt 6	Experiment	Random	Phantom	Real
	VAS (Pre/Post)	83 / 85	87 / 88	84 / 80
	SF-MPQ2 (Pre/Post)	65 / 75	77 / 87	110 / 86
Pt 7	Experiment	Random	Phantom	Real
	VAS (Pre/Post)	41 / 40	78 / 87	68 / 61
	SF-MPQ2 (Pre/Post)	7 / 9	44 / 49	46 / 43
Pt 8	Experiment	Random	Phantom	Real

	VAS (Pre/Post)	21 / 23	14 / 24	30 / 28
	SF-MPQ2 (Pre/Post)	7 / 12	10 / 8	20 / 18
Pt 9	Experiment	Phantom	Random	Real
	VAS (Pre/Post)	43 / 64	15 / 26	19 / 18
	SF-MPQ2 (Pre/Post)	27 / 40	8 / 9	6 / 6
Pt 10	Experiment	Phantom	Random	Real
	VAS (Pre/Post)	12/13	23/21	22/22
	SF-MPQ2 (Pre/Post)	4/4	16/16	12/11

VAS: Visual Analog Scale; SF-MPQ2: Short-form McGill Pain Questionnaire 2; Phantom: Experiment with phantom decoder; Random: Experiment with random decoder; Real: Experiment with real decoder.
